# An Innovative Olive Pâté with Nutraceutical Properties

**DOI:** 10.3390/antiox9070581

**Published:** 2020-07-03

**Authors:** Pierpaolo Cavallo, Irene Dini, Immacolata Sepe, Gennaro Galasso, Francesca Luisa Fedele, Andrea Sicari, Sergio Bolletti Censi, Anna Gaspari, Alberto Ritieni, Matteo Lorito, Francesco Vinale

**Affiliations:** 1 Dipartimento di Fisica, Università di Salerno, Via Giovanni Paolo II, 132, 84084 Fisciano, Salerno, Italy; 2 ISC-CNR, Institute for Complex Systems, Via dei Taurini, 19, 00185 Roma, Italy; 3 Dipartimento di Farmacia, Università degli Studi di Napoli “Federico II”, 80131 Napoli, Italy; annagaspari@virgilio.it (A.G.); alberto.ritieni@unina.it (A.R.); 4 Diagnostica Cavallo—Centro Ricerca Albo MIUR, 84123 Salerno, Italy; ricerca@cavallo.net; 5 Dipartimento di Medicina e Farmacia, Università di Salerno, 84081 Baronissi, Salerno, Italy; ggalasso@unisa.it; 6 Linfa Scarl, University Spin Off, 80146 Napoli, Italy; francalisa@yahoo.com (F.L.F.); andrea@laboratoriolinfa.it (A.S.); sergio@laboratoriolinfa.it (S.B.C.); 7 UNESCO Chair of Health Education and Sustainable Development, University of Naples, 80131 Napoli, Italy; 8 Dipartimento di Agraria, Università di Napoli “Federico II”, 80055 Portici (NA), Italy; lorito@unina.it; 9 Dipartimento di Medicina Veterinaria e Produzioni Animali, Università degli Studi di Napoli “Federico II”, 80137 Napoli, Italy; frvinale@unina.it

**Keywords:** olive mill wastewater, olive oil, Olive Pâté, antioxidants, nutraceutical, Q Exactive Orbitrap LC-MS/MS

## Abstract

Food plays a central role in health, especially through consumption of plant-derived foods. Functional foods, supplements, and nutraceuticals are increasingly entering the market to respond to consumer demand for healthy products. They are foods, supplements, and ingredients which offer health benefits beyond the standard nutritional value. Some benefits are associated with phenolic compounds and phytochemicals with antioxidant properties. An olive pâté (OP) was added with antioxidants derived from olive mill wastewater (OMWW) to obtain a functional product rich in phenolic compounds. The olive pâté is produced from the ground olive pericarp, which shows an excellent natural antioxidant content. The OMWW is a waste product from oil processing, which is also rich in phenolic compounds. The result was a product rich in trans-resveratrol, OH tyrosol, and tyrosol in concentrations such as satisfying the European community’s claims regarding the possible antioxidant action on plasma lipids with excellent shelf-life stability. The total phenolic content was assayed by a colorimetric method, the antioxidant activity by the ABTS [(2,2′-azino-bis (3-ethylbenzothiazoline-6-sulfonic acid)] test, the phenolic profile by Q Exactive Orbitrap LC-MS/MS. The shelf-life stability was confirmed by yeast, molds, and total microbial count, pH, and water activity determinations, and the best pasteurization parameters were determined. The palatability was judged as excellent.

## 1. Introduction

Extra virgin olive oil (EVOO) is one of the highly valued products of the so-called ‘Mediterranean Diet’. A diet indicated by Ancel Keys in the 1980s inspired by the eating habits of Italy and Greece in the 1960s. This diet suggests high consumption of olive oil, unrefined cereals, legumes, fish, vegetables and fruits, and moderate consumption of cheese, meat, and wine, to reduce the risk of some chronic diseases such as cardiovascular and cancer diseases [1]. The health-promoting action of the EVOO is linked to the phenolic fractions profile, which includes tyrosol (tyr), hydroxytyrosol (OH tyrosol), secoiridoids, and lignans [2].

The EVOO production includes washing of olives, followed by their crushing in a hammer mill to obtain ‘pomace’, that is a mixture of the crushed olive pericarp and stone; the pomace is then pressed to extract a liquid mix of oil and water, the olive mill wastewater (OMWW), which are finally separated to obtain the clear oil. The OMWW is rich in natural antioxidants. It contains 53% of the olives phenolic fraction [3,4]. These molecules can be recovered from OMWW with different methods [3,5,6,7]. Conventional separation techniques used for this purpose are the chromatography, extraction, centrifugation, and membrane separation.

New technologies used to decrease energy consumption and increase the extraction efficiency are electro-technologies (high voltages electrical and discharges pulsed electric fields), ultrasounds, microwaves, mechanical technologies (pressurized liquid extraction), extraction techniques with supercritical fluids, and filtration methods with reverse osmosis and tangential ultrafiltration systems. [8]. The phenolic fraction recovered from OMWW may be used to improve the content of antioxidants in foods. This “reinforcement” is extensively studied [9,10,11,12,13,14].

In this study, an OMWW concentrated was added to OP, to obtain a reinforced olive pâté (ROP) rich in phenols. A new olive-derived nutraceutical product, made of natural ingredients, capable of supplementing the antioxidants in the diet, without changing dietary habits. Olive pâté (OP), sometimes named ‘olive fruit paste’, is an olive-derived food of the traditional Mediterranean gastronomy with a coarse texture. The OP main ingredients are more or less finely ground [15] prepared using only the pericarp; pâté differs from pomace, since the latter is made from pericarp and stone of the olives, and it is not appropriate for gastronomic use. OP can include other ingredients—like garlic, capers, etc.—and usually contains olive oil as a covering agent. OP is used as a condiment for pasta, hard-boiled eggs, salads, snacks, etc., and is growingly being recognized [16] not only for its gastronomic role, but also by its beneficial effects on human health.

Several studies are mainly focused on the chemical/microbiological characteristics and the sensory properties of OP [15,16,17,18,19,20,21]. No data describe the functional properties and the stability of OP products added with OMWW. The nutraceutical potential of the ROP was evaluated in terms of the total phenol content, antioxidant activity, and phenolic compound profile. The shelf-life of ROP was also tested.

## 2. Materials and Methods

### 2.1. OP and ROP Preparation

**Figure 1:**
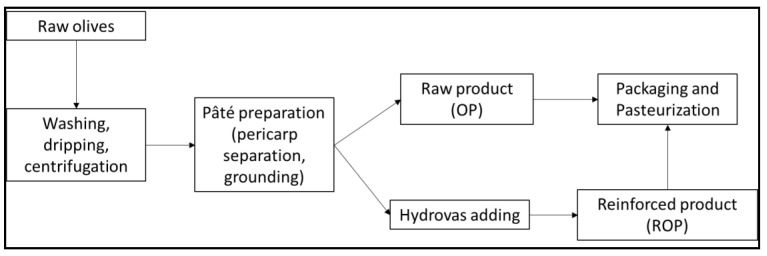
Reinforced olive pâté production process.

The commercial OMWN extract of *Olea europea* fruit was from Hydrovas 10 (Bionap, Belpasso, Italy), The OP preparation, resumed in Figure 1, included washing, separation of pericarp from stone, smashing and mixing with olive oil, packaging, and pasteurization.

The ROP preparation was made by adding Hydrovas to the raw OP, to obtain two different final concentrations. The two concentration levels were 100 and 200 mg of phenols for 20 g of product, called 2X (10) and 4X (20) reinforced olive pâtés (2X ROP and 4X ROP), respectively.

### 2.2. Pasteurization Process

Pasteurization of OP and ROP was performed in a tunnel pasteurizer (TECNINOX, Parma, Italy) on the product previously packaged in glass containers. The thermal action was applied to the container, cap, or lid. The temperature of the box was raised with hot water showers at controlled temperatures and maintained for the time required for the pasteurization cycle. Subsequently, it was gradually lowered with water showers at decreasing temperature. Three different combinations of time and temperature: 86 °C for 20 min, 90 °C for 15 min, and 94 °C for 10 min were used.

### 2.3. Chemicals

Luteolin (≥98%; CAS number 491-70-3); apigenin (≥95%; CAS number 520-36-5); trans resveratrol (CAS number 501-36-0); oleuropein (≥98%; CAS number 36619-42-4); verbascoside (≥99%; CAS number 61276-17-3); isoverbascoside (≥95%; CAS number 61303-13-7); tyrosol (≥95%; CAS number 501-94-0); vanillic acid (≥97%; CAS number 121-34-6); cinnamic acid (≥99%; CAS number 140-10-3); ferulic acid (≥99%; CAS number 537-98-4); p-coumaric acid (≥98%; CAS number 501-98-4); 4-hydroxybenzoic acid (≥99%; CAS number 99-96-7); 3-hydroxybenzoic acid (≥99%; CAS number 99-06-9) were purchased from Sigma Aldrich (St. Louis, MO, USA); secologanoside (>95%) were bought from ChemFaces Biochemical Co., Ltd. (Hubei, China); hydroxytyrosol (>95%) were obtained by Indofine (Hillsborough, NJ, USA); oleuropein-aglycone monoaldehyde (>95%) were purchased from Extrasynthese (Genay, France). Ligstroside were purchased from Wuhan Golden Wing Industry & Trade Co., Ltd. (Wuhan, China), the ligstroside-aglycone monoaldehyde (>95%) was isolated by chromatographic method and identity by nuclear magnetic resonance (NMR). The other chemicals were the analytical (Sigma Aldrich, St. Louis, MO, USA).

### 2.4. Total Phenolic Compounds

The total phenol content was determined colorimetrically at 765 nm, using the Folin–Ciocalteau reagent Sigma Aldrich (St. Louis, MO, USA) as described by Singleton and coll [22]. 10 mL of a methanol/water solution (80:20 *v/v*) and Tween 20 (2% *v/v*) was mixed with 10 g of OMWW; the mixture was homogenized with the ULTRA-TURRAX^®^ (Ika, Breisgau, Germania) at 25 °C to 15,000 RPM; then the homogenate was centrifuged for 10 min at 5000 RPM. The supernatant was collected and placed in the freezer for 24 h at −20 °C [23,24,25,26,27,28]. A Falcon (15 mL) was shaken by 2.5 mL H_2_O, 625 μL methanolic extract, 625 μL of Folin-Ciocalteau’s phenol reagent and, after 6 min, 6.25 mL of 7% Na_2_CO_3_ and 5 mL dd H_2_O. After incubation for 90 min at room temperature, the absorbance of the reagent blank was determined at 760 nm by spectrophotometer (V-530 Jasco, Tokyo, Japan). The total concentration of polyphenols was expressed as mg/L of gallic acid. The determination of the polyphenol content was made both on unpasteurized and on heat-treated samples. All samples were analyzed in triplicate.

### 2.5. Antioxidant Activity Determination

The antioxidant activity was evaluated with ABTS colorimetric method [29]. A stock solution was obtained dissolving 9.6 mg of ABTS in 2.5 mL of water and adding 44 mL of a solution made by dissolving 37.5 mg of K_2_S_2_O_8_, in 1 mL of water. The stock solution was kept in the dark at 4 °C for 8 h before use. The work solution was obtained from the stock solution by dilution using a 1:88 (*v/v*) ratio (it must measure between 0.7 and 0. 8 at 734 nm). Subsequently, 100 μL of sample and 1 mL of work solution were added, and A734 was measured exactly after 2 min and 30 s. (V-530 Jasco, Tokyo, Japan). The calibration curve was obtained using Trolox (Sigma Aldrich—St. Louis, MO, USA), and results were expressed as mmol Trolox/100 g. All biological samples were analyzed in triplicate.

### 2.6. UHPLC Operative Condition

An Agilent Technologies 1200 Series Ultra High Liquid Chromatograph (UHPLC) (Agilent, Santa Clara, CA, USA) equipped with pre-column Phenomenex (Torrance, CA, USA), and column Accucore aQ 2.6 µm 100 × 2.1 mm Thermo Scientific (Waltham, MA, USA) was used for experimental purposes. The injection volume was 5 μL. A gradient was employed as a mobile phase (Table 1).

### 2.7. Orbitrap UHPLC-MS/MS Operative Condition

All mass experiments were conducted at a Q Exactive Orbitrap LC-MS/MS (Thermo Fisher Scientific, Waltham, MA, USA) equipped with an ESI source (HESI II, Thermo Fisher Scientific, Waltham, MA, USA) operating in negative ion mode (ESI-). (Table 2) The accuracy and calibration of the Q Exactive Orbitrap LC-MS/MS were checked weekly. A reference standard mixture was purchased by Thermo Fisher Scientific (Waltham, MA, USA). The Xcalibur software v. 3.1.66.10 (Xcalibur, Thermo Fisher Scientific, Waltham, MA, USA) was used to analyze and process data.

### 2.8. Shelf-Life Analysis

#### 2.8.1. Microbiological Analysis

The total microbial count was enumerated on non-selective medium Plate Count Agar (PCA, Oxoid, Milan, Italy). Using a sterile procedure, 1 mg of sample was diluted with 9 mL of physiological saline (9 g/L NaCl) and were prepared serial dilution. The total microbial count was determined by pour plating of suitable dilution on with 15–18 mL of PCA medium and incubated at 30 °C for 48 h.

Yeast and Molds (YM) were enumerated on selective medium Sabouraud Dextrose Agar (SDA, Oxoid, Milan, Italy). The plates were incubated at 25 °C for 5 days. The number of bacteria and YM were expressed as colony forming units (CFU), at three different temperatures: 25, 37, and 55 °C.

#### 2.8.2. pH and Water Activity

The pH of the olive pâté was measured using pH meter HI 9025 (Hanna Instruments, Woonsocket, RI, USA). 10 g samples were diluted in 10 mL of distilled water and were centrifuged for five minutes at 6000 RPM. The supernatant was filtered with a 45 µm Millex membrane (Merk, Darmstadt, Germany).

Water was measured using a water activity system TESTO 650 (Testo SpA, Milan, Italy).

#### 2.8.3. Stress Test and Pasteurization Effects

The technological stability of the olive pâté was evaluated with the stress test. Two different types of samples (commercial and reinforced) were subjected to different temperatures (25, 37, 55 °C) for 19 days, and then tested at days 0, 2, 7, 14, 16, and 19 for pH and antioxidant activity, using the methods listed before.

Pasteurization effects were evaluated by assaying total phenols and antioxidant activity in samples of OP and in the two levels of ROP prepared (2X and 4X) treating them at three different combinations of time and temperature: 86 °C for 20 min, 90 °C for 15 min, and 94 °C for 10 min.

### 2.9. Palatability Test

The palatability of the two different olive pâté products, OP (commercial), and ROP (reinforced) was tested.

A spontaneous panel was formed by the researcher team members, who tasted 3.5 mL of the two products at room temperature, giving a judgement on a 1–10 scale (1 = worst, 10 = best) about texture, appearance, smell, taste, and after taste.

### 2.10. Statistical Analysis

Excel spreadsheet software, version 19.0 (Microsoft, Redmond, WA, USA) was used to perform statistical analyses.

## 3. Results

### 3.1. Phenolic Profile and Concentrations

A UHPLC-MS/MS method was employed to delineate the phenolic profile and dosage.

Eighteen phenolics—including three flavonoids, six phenolic acids, seven secoiridoids, and two phenolic alcohols—were characterized and dosed. Table S1, in the supplementary data, reported the chromatographic and spectroscopic parameters used to identify phenolics in samples. The quantification method was validated (AOAC, 2012) [30]. The matrix effect (ME, signal enhancement or suppression) was investigated by calculating the ratio percentage between the slopes of the matrix-matched calibration curve and the curve in solvent (Figure S1). The linearity was guaranteed by the coefficient of regression of the calibration curve close to 1. LODs and the LOQs range verified the sensitivity, triplicate injection of each phenolic standard, at seven different concentrations confirmed the intraday repeatability.

**Figure 2:**
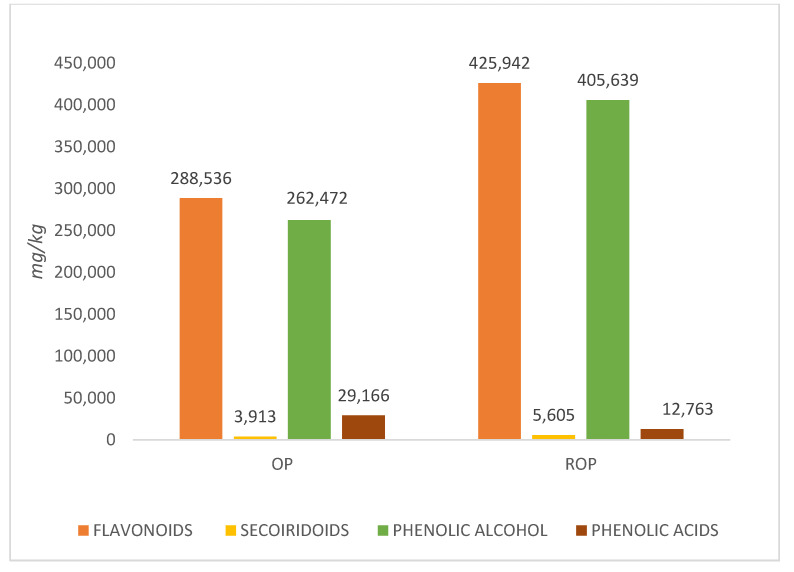
Total concentration of each class of phenolic compounds.

Flavonoids were the most representative phenolics in the OP and ROP samples, following by phenolic alcohols and then all the others (Table 3, Figure 2). The highest concentration in the raw OP was presented by *trans*-resveratrol, HT, and tyrosol, followed by vanillic acid, luteolin, 3-OH benzoic acid, secologanoside, and p-coumaric acid. The highest increment in concentration, 269.7% was presented by ligstroside-aglycone monoaldehyde, followed by apigenin with 96.4%, but both had a low initial level. The most significant increments were presented by molecules with high initial concentration, which were luteolin, more than doubled; tyrosol, 69.3% higher of baseline; *trans*-resveratrol, and HT, both over 40% of baseline. Only the phenolic acids were higher in OP than ROP.

The HPLC profiles of OP and ROP are reported in the Supplementary Data.

The total phenols concentrations were tested by the colorimetric method ≅765 nm, with the results expressed in mg/L of gallic acid; the results were OP = 2620 and ROP = 4750, with an increment of 81.2%. The antioxidant activity was tested by ABTS, with the results expressed in µmol/100 g of TROLOX; the results were OP = 715,131 and ROP = 975,951, with an increment of 36.4%.

**Figure 3:**
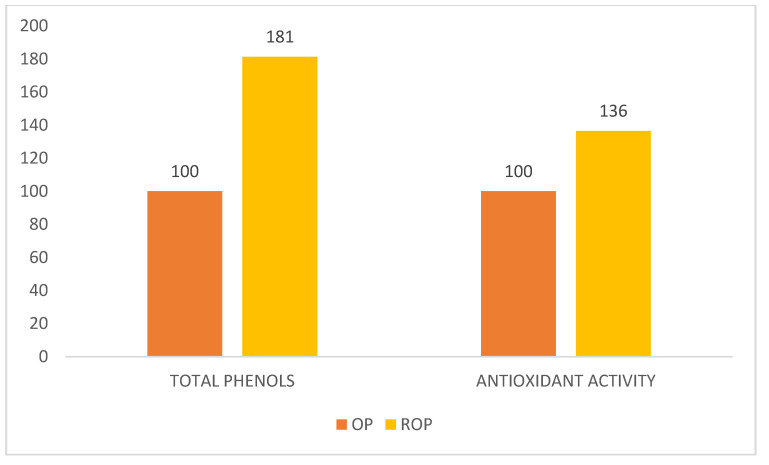
Comparison of the reinforcement effects (OP = 100).

To graphically compare the effects of reinforcement, in Figure 3 we present the data after normalization; the y axis shows this index number, given the OP base value at 100.

It is clearly appreciable the higher effect of reinforcement on phenols rather than antioxidant activity.

### 3.2. Other Analytical Results

For pH and aW, OP and ROP had the same values, with pH = 4.39 tested by the potentiometric method, and aW = 0.91 measured in a sealed container.

The Total microbial and the Mold/yeast counts were negative for any kind of growth for both OP and ROP.

**Figure 4:**
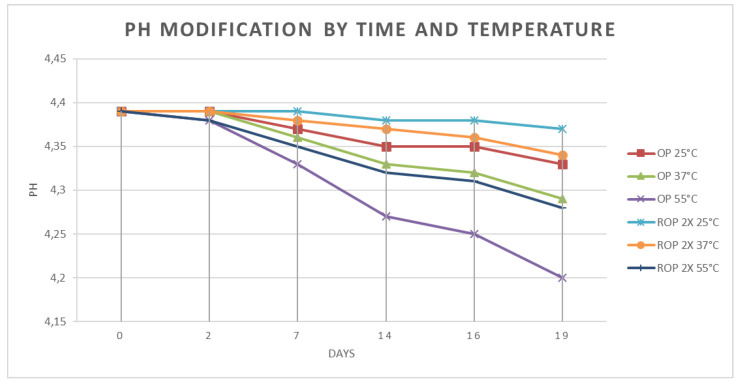
Results of the stress test for pH.

The stress tests results for pH are shown in Figure 4 Starting from the same initial value of 4.39, OP and ROP underwent a slight decrease, that was different for each product and condition of stress. The ROP obtained the best performance at 25 °C, which finished with a value of 4.37 at 19 days, while the worst result was obtained at 55 °C, even if with a pH reduction limited at 0.09 for the OP, with a final value at 4.28.

**Figure 5:**
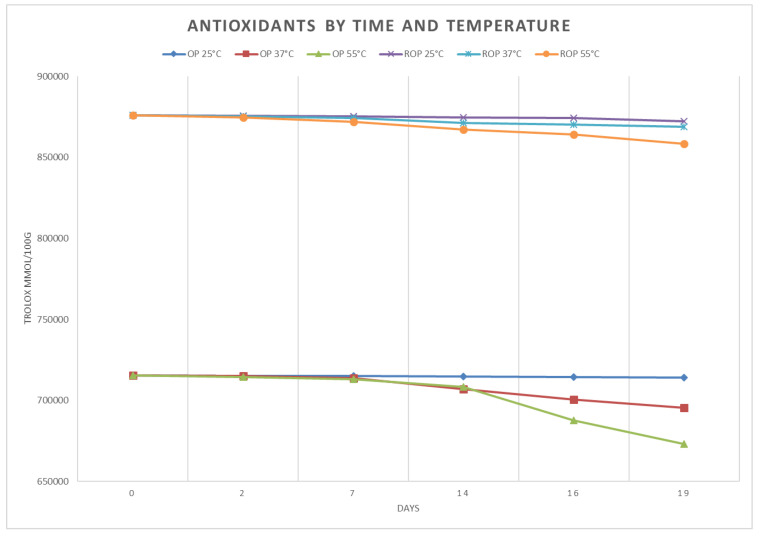
Results of the stress test for antioxidants.

The stress tests results for antioxidant activity are shown in Figure 5 The ROP started from higher levels, and showed lower degradation than OP. Anyway, the maximum degradation obtained at 19 days was limited, as OP lost about 6% while ROP about 2% of the initial activity. For both, a significant degradation was shown only at the highest temperature of 55 °C.

The pasteurization effects were evaluated comparing three different combinations of time and temperature (t/t): 86 °C for 20 min, 90 °C for 15 min, and 94 °C for 10 min. 

The contents of phenols expressed as Gallic acid, and antioxidants as TROLOX are reported in Table 4, comparing the pasteurized products with the raw, either in absolute values and in form of Index, considering the raw product = 100.

The proportion of phenols and for antioxidants remaining after pasteurization was higher for ROP than OP, with a highly significant statistical significance (*p* < 0.01).

The palatability test gave excellent result: the average overall score was 9/10 for the OP, and 8.5/10 for the ROP. In detail, the scores for texture, appearance and smell were all excellent for both the products, while taste for ROP was equally considered excellent by those who appreciated its slightly soreness but a bit less by those who did not, and after taste perception followed the same judgment as the taste.

## 4. Discussion

The olive oil market is constantly growing, as it is considered a healthy product.

Unfortunately, oil production leads to the formation of environmentally harmful waste: solid and liquid residues are obtained with a high organic weight detrimental to the environment, and their natural residues vary according to the system used to extract the olive oil. For example, centrifugation systems (two-phase and three-phase) produce extra virgin olive oil, olive pomace (solid residue), and olive vegetation water (liquid residue).

Olives contain polyphenols [31,32], biomolecules with antioxidant activity able to decrease the risk of cancer, cardiovascular diseases, and inflammation [33,34,35,36]. Polyphenols are amphiphilic molecules, some of which are more soluble in water, others in oil; therefore, some classes are more concentrated in the extra virgin olive oil, others in the vegetation water fraction. Phenols are generally used to produce nutraceuticals, functional foods, and food additives [37,38,39].

In the present work, an olive pâté (OP) reinforced with concentrated OMWW was studied to obtain a nutraceutical product with a high phenol content and long shelf life. Olive pâté used in the Mediterranean diet is an olive fruit paste, made by pericarp only. For centuries, olive pâté has been produced only in small quantities, usually handcrafted, according to ethnic traditions of the Mediterranean area.

The process of reinforcement of foods with micronutrients is well established and widespread, with a set of WHO-FAO Guidelines [40] that have defined it. Some have concerned refined olive oil [41], and no specific experience appears to have been made with olive pâté even if are present experiences of fortification with phenols in many different foods.

Results showed that the addition of OMWW to OP strongly increased the total phenol content of ROP (81.2%), and little the antioxidant potential (36.4%). The slightest variation in the antioxidant activity may depend on the method used, as ABTS is known to underestimate the antioxidant potential of phenolic compounds with a complex structure and size, because the ring adducts—mainly secondary rings (Group 3)—interfere with phenol access to the ABTS•+, decreasing the reaction efficiency every OH added [42].

The inhomogeneous composition of the different classes of phenols is noteworthy; in particular, the very abundant concentration of trans-resveratrol, followed by OH tyrosol and tyrosol in ROP. In nature, resveratrol occurs in two stereoisomer forms: cis- and trans. The trans isomer is biologically more active than cis [43].

Resveratrol has antioxidant, coronary vasodilating, antihypertensive, neuroprotective, anti-inflammatory, and anticancer properties [44]. In vivo studies have demonstrated low bioavailability after oral intake. Therefore, the development of nutraceutical formulations with better pharmacologic properties is an exciting task. Resveratrol-enriched foods would allow a higher daily intake of resveratrol (therapeutically doses are ~1 g) than conventional foods [44]. Red wine is usually considered a good source of resveratrol, generally containing 0.361–1.972 mg/L, while ROP 404.388 mg/Kg fresh weight [45].

Moreover, ROP is an interesting source of the OH tyrosol (204.5102 mg/Kg) and tyrosol (201.129 mg/Kg), which have shown antidiabetic, anti-obesity, cardioprotective, antiatherogenic, neuroprotective, anticancer effects [46]. In ROP 2X, there is a concentration of OH-tyrosol (8.1 mg/20 g) higher than that required by EFSA (5 mg/20 g) to be able to attribute CLAIM “oil polyphenols contribute to the protection of blood lipids from oxidative stress” to olive oil (EU 432/2012).

OP and ROP were subjected to a pasteurization process to guarantee their safety, increase hygienic stability, interrupting the oxidative process, and extend their shelf life [47]. When the pasteurization process uses high temperature in short-time, bacterial spoilage is the most limiting factor in extending the shelf life of the processed products [48]. Aroma and taste attributes of the products change when microorganism’s growth and consequential decrease consumer acceptability of the products. In this work, the microbial, mold, and yeast stability were checked, and no microorganisms were found to grow. This was partly due to the presence of high concentrations of phenols with antibacterial properties [25,26]. Moreover, the growth of bacteria surviving the pasteurization process was tested by monitoring the changes in pH [49].

The stability test for pH showed different performances between OP and ROP: the most stable preparation was ROP, with minimal reduction, from 4.37 to 4.36 for the worst storage case, 55 °C for 19 days, while in the same condition the OP showed a higher, even if still limited, variation, from 4.37 to 4.28.

Previous studies have shown that pasteurization can lead to loss of the antioxidant activity since phytochemicals with antioxidant properties are affected differently by heat treatments [50]. The stability test for antioxidant activity showed different performances between the two products, as ROP started from higher levels and showed a limited loss, about 2%, for the worst storage case, 55 °C for 19 days, while in the same condition the OP showed a higher, even if still limited, antioxidant activity loss of 6%. The reason may be referred to as the protective action of olive antioxidants on the stability of food [51].

We evaluated which pasteurization process guaranteed the best results to minimize the losses of phenolic compounds and antioxidant activity. The best performance was achieved pasteurizing at 90 °C for 15 min. In this condition, total phenols concentration was 95%, and antioxidant activity was 32.9%, higher in the ROP than OP product proving that the reinforcement process changes the biochemical characteristics of the olive pâté apart from enhancing its nutraceutical properties.

Finally, the excellent palatability of the ROP in comparison with the OP may suggest that this product, and probably other products of the same kind, are going to be well accepted by the healthy food market, and will have interesting commercial perspectives as they widen the possibilities of use of olive derived products in nearly any kind of diet.

## 5. Conclusions

In this study, we tested the possibility of producing a new nutraceutical product based on olive pâté and OMWW. We obtained a product rich in trans resveratrol, OH tyrosol, and tyrosol in concentrations such as to satisfy the claims of the European community regarding the possible antioxidant action on plasma lipids. The product shows good palatability, and good results in terms of stability, thus having an interesting market perspective either from both nutraceutical and commercial points of view.

## Figures and Tables

**Table 1:** UHPLC mobile phase.

Minutes	Phase B% 100% Acetonitrile(ACN)	Phase A% H_2_O Containing 0.1% (*v/v*) Acetic Acid (AcH)
0–5	5	95
6–25	40	60
25.1–27	100	0
27.1–35	5	95
35.1–45	0	100

**Table 2:** Mass operative setting.

Ion Source Parameters	
Spray Voltage	−3.0 kV
Sheath gas	(N_2_ > 95%) 30
Auxiliary gas	(N_2_ > 95%) 15
Capillary temperature	200 °C
S-lens	RF level 50
Auxiliary gas	305 °C
**Analyzer Target SIM (Single Ion Monitoring) Parameter**	
Automatic gain control (AGC)	target set at 1 and 6
Resolution	140,000 FWHM (full width at half maximum)
Scan rate	(100–500 *m/z*)

**Table 3:** Phenolic compounds in OP and ROP.

Group	Compound	OP (mg/kg)	ROP (mg/kg)	Delta	Delta% of Baseline
Flavinoids	*trans*-Resveratrol	278.1 ± 2.0	404.4 ± 10.2	126.2	45.4%
	Luteolin	9.9 ± 0.09	20.6 ± 0.1	10.7	107.8%
	Apigenin	0.5 ± 0.0	0.9 ± 0.0	0.45	96.4%
Secoiridoids	Ligstroside	0.2 ± 0.004	0.2 ± 0.004	0.038	20.5%
	Secologanoside	1.6 ± 0.016	1.9 ± 0.019	0.251	15.4%
	Verbascoside	0.6 ± 0.002	0.8 ± 0.009	0.186	31.1%
	Isoverbascoside	0.2 ± 0.004	0.4 ± 0.003	0.139	55.8%
	Oleuropein-aglycone monoaldehyde	0.5± 0.008	0.7 ± 0.011	0.156	30.6%
	Ligstroside-aglycone monoaldehyde	0.3 ± 0.026	1.2 ± 0.031	0.88	269.9%
	Oleuropein	0.4 ± 0.010	0.4 ± 0.002	0.042	10.2%
Phenolic Alcohol	Tyrosol	120.8 ± 6.6	204.5 ± 5.9	83.6	69.3%
	OH-Tyrosol	141.7± 3.1	201.1 ± 4.01	59.4	42.0%
Phenolic Acids	P-Coumaric	1.1 ± 0.014	0.5 ± 0.011	−0.619	−56.1%
	Cyinnamic	0.5 ± 0.017	0.01 ± 0.057	−0.522	−98.9%
	Ferulic	0.3 ± 0.002	0.06 ± 0.001	−0.25	−81.4%
	Vanillic	21.8 ± 0.055	9.0 ± 0.077	−12.7	−58.8%
	4-Hydroxybenzoic acid	1.0 ± 0.002	0.4 ± 0.007	−0.555	−57.8%
	3-Hydroxybenzoic acid	4.5 ± 0.061	2.8 ± 0.064	−1.66	−36.9%

OP = olive paté; ROP = reinforced olive pâté; Delta = absolute difference; Delta% of baseline = difference in per cent of the OP value.

**Table 4:** Antioxidant activities after pasteurization under different t/t combinations.

Parameter	Pasteurization Combinations			
	Raw	86 °C/20′	90 °C/15′	94 °C/10′
OP-TROLOX µmol/100 g	715.31	455.308	515.308	435.308
ROP-TROLOX µmol/100 g	875.951	620.286	685.286	597.286
OP-Gallic Acid mg/L	2620	1820	1340	1520
ROP-Gallic Acid mg/L	4750	3550	2700	2850
OP-Index for TROLOX (Raw = 100)	100	63.7	72.1	60.9
ROP-Index for TROLOX (Raw = 100)	100	70.8	78.2	68.2
OP-Index for Gallic Acid (Raw = 100)	100	69.5	51.1	58.0
ROP-Index for Gallic Acid (Raw = 100)	100	74.7	56.8	60.0

## Supplementary Materials

The following are available online at https://www.mdpi.com/2076-3921/9/7/581/s1, Figure S1: HPLC profiles of reinforced olive pâté (ROP-Upside chromatogram) and olive pâté (OP–Down side chromatogram); Table S1: Validation parameters of the UHPLC- MS/MS method of analysis.
