# Supplementary material for: An Innovative Olive Pâté with Nutraceutical Properties
Source: Antioxidants (Basel). 2020 Jul 3;9(7):581. doi: 10.3390/antiox9070581 (PMC7401864; doi:10.3390/antiox9070581)
Supplement: Supplementary file 1 [file antioxidants-09-00581-s001.pdf]

Supplementary data

Table S1. Validation parameters of the UHPLC- MS/MS method of analysis.

Group	Compound	Linearity *(mg/Kg)*	R^2	LOD *(mg/Kg)*	LOQ *(mg/Kg)*	ME (%)	Intraday RSD *% 50 mg/Kg*	*RT (min)*	*Formula*	*Theoretical m/z [M – H]-*	*Experimental m/z of [M – H]-*	*Calculated Errors Δppm*	*Fragments*	Collision Energy *(eV)*
	Luteolin	0.5–50	0.991	0.066	0.200	85	1.4	19.07	$C_{15}H_{10}O_6$	285.04046	285.04106	2.10	133.02940	30
FLAVONOIDS	Apigenin	0.5-50	0.899	0.066	0.800	92	2.1	19.12	$C_{15}H_{10}O_5$	269.04555	269.04597	1.56	225.05592	35
	trans Resveratrol	0.5-5.0	0.898	0.090	0.200	98	1.8	16.65	$C_{14}H_{12}O_3$	227.07137	227.07147	0.44	185.06082	30
	Oleuropein	1-50	0.991	0.166	0.500	102	5.0	16.69	$C_{25}H_{32}O_{13}$	539.17701	539.17767	1.22	377.12393	20
	Verbascoside	1-50	0.88	0.163	0.500	87	1.3	14.67	$C_{29}H_{36}O_{15}$	623.19814	623.19866	0.83	161.02362	15
	Ioverbascoside	1-50	0.89	0.716	0.500	109	1.4	15.49	$C_{29}H_{36}O_{15}$	623.19814	623.19854	0.64	161.02362	15
SECOIRIDOIDS	Ligstroside	1-50	0.991	0.166	0.500	105	4.0	18.25	$C_{25}H_{32}O_{12}$	523.18210	523.18279	1.32	361.12914	12
	Secologanoside	1-50	0.967	0.333	1.000	94	2.1	19.49	$C_{16}H_{21}O_{11}$	389.1092	389.109258	0.59	345.1195	12
	Oleuropein-aglycone monoaldehyde	1-50	0.998	1.000	3.000	88	2.1	21.25	$C_{19}H_{22}O_8$	377.12419	377.12442	0.61	345.09790	12
	Ligstroside-aglycone monoaldehyde	1–50	0.999	0.033	0.100	91	0.7	21.59	$C_{19}H_{22}0_7$	361.12145	361.12141	-0.11	291.1122	21
PHENOLIC ALCOHOL	Tyrosol	1-50	0.991	0.133	0.040	107	1.6	2.75	$C_8H_{10}O_2$	137.06080	137.06096	1.17	119.05022	12
	Hydroxytyrosol	1-50	0.992	0.666	2.000	95	3.0	1.60	$C_8H_{10}O_3$	153.05572	153.05580	0.52	123.04561	12
	Vanillic acid	1-50	0.887	0.200	0.600	101	1.1	4.30	$C_8H_8O_4$	167.03498	167.03522	1.44	152.01143	20
	Cinnamic acid	1-50	0.991	0.200	0.600	96	0.9	11.54	$C_9H_8O_2$	147.04515	147.04536	1.43	103.04501	20
PHENOLIC ACIDS	Ferulic acid	1-50	0.912	0.100	0.300	81	1.7	11.81	$C_{10}H_{10}O_4$	193.05063	193.05084	1.09	178.02685	20
	p-Coumaric acid	1-50	1.000	0.100	0.300	97	1.8	9.71	$C_9H_{10}O_5$	163.04007	163.04028	1.29	119.05023	20
	4-Hydroxybenzoic acid	1-50	0.998	0.207	0.622	104	0.9	2.57	$C_7H_6O_3$	137.02442	137.02456	1.02	93.03431	12
	3-Hydroxybenzoic acid	1-50	0.995	0.205	0.622	112	1.1	2.88	$C_7H_6O_3$	137.02442	137.02458	1.17	93.03431	12

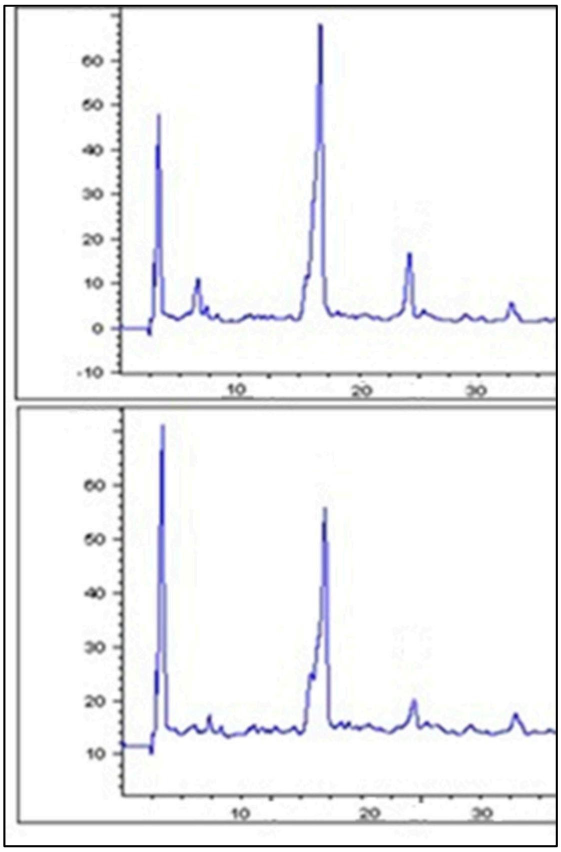

Figure S1. HPLC profiles of Reinforced Olive Pâté (ROP - Up side chromatogram) and Olive Pâté (OP – Down side chromatogram).
